# Diabetes mellitus is independently associated with adverse clinical outcome in soft tissue sarcoma patients

**DOI:** 10.1038/s41598-020-69237-y

**Published:** 2020-07-24

**Authors:** Adrian Stelzl, Faisal Aziz, Jakob M. Riedl, Florian Posch, Maria A. Smolle, Tatjana Stojakovic, Angelika Terbuch, Martin Pichler, Marko Bergovec, Andreas Leithner, Bernadette Liegl-Atzwanger, Michael Stotz, Armin Gerger, Harald Sourij, Joanna Szkandera

**Affiliations:** 1 Division of Clinical Oncology, Department of Medicine, Medical University of Graz, Auenbruggerplatz 15, 8036 Graz, Austria; 2 Division of Endocrinology and Diabetology, Department of Medicine, Medical University of Graz, Graz, Austria; 3 Center for Biomarker Research in Medicine, CBmed, Graz, Austria; 4 Department of Orthopedics and Trauma, Medical University of Graz, Graz, Austria; 5 Clinical Institute of Medical and Chemical Laboratory Diagnostics, University Hospital Graz, Graz, Austria; 6 Research Unit Genetic Epidemiology and Pharmacogenetics, Division of Clinical Oncology, Medical University of Graz, Auenbruggerplatz 15, 8036 Graz, Austria; 7 Institute of Pathology, Medical University of Graz, Graz, Austria

**Keywords:** Sarcoma, Diabetes

## Abstract

Diabetes mellitus (DM) and hyperglycemia are known predictors of adverse outcome in different tumor entities. The present study investigated the effect of DM and pre-surgery blood glucose levels on cancer specific survival (CSS), overall survival (OS), and disease-free survival (DFS) in non-metastatic soft tissue sarcoma (STS) patients. A total of 475 STS patients who underwent curative resection were included in this retrospective study. CSS, DFS, and OS were assessed using Kaplan–Meier curves. The association between pre-existing DM as well as mean pre-surgery blood glucose levels and all 3 survival endpoints was analyzed using Cox-hazard proportional (for OS and DFS) and competing risk regression models (for CSS). In unadjusted analysis, DM was significantly associated with adverse CSS (sub-hazard ratio [SHR]: 2.14, 95% confidence interval [CI] 1.18–3.90, p = 0.013) and OS (hazard ratio [HR]: 2.05, 95% CI 1.28–3.28) and remained significant after adjusting for established prognostic factors (CSS: adjusted SHR 2.33, 95% CI 1.21–4.49, p = 0.012; OS: adjusted HR 1.96, 95% CI 1.17–3.28, p = 0.010), respectively. There was no significant association of DM with DFS (p = 0.149). The mean pre-surgery glucose levels were not significantly associated with inferior outcome (CSS: p = 0.510, OS: p = 0.382 and DFS: p = 0.786). This study shows, that DM represents a negative prognostic factor for clinical outcome in STS patients after curative resection.

## Introduction

STS are rare malignant tumors of mesenchymal origin with heterogeneous and complex histopathology^1^. Approximately 50% of patients with localized high-grade STS develop local recurrence or distant metastases, even after tumor resection with curative intent^2^. Therefore, it is important to identify patients who are at high risk for local tumor recurrence and dissemination. There is a variety of established prognostic factors in STS. These include clinical as well as histopathological findings such as age at diagnosis, tumor size, histologic tumor grading, histologic subtype, tumor depth, tumor site, and margin status^2,3^. To predict sarcoma specific survival, OS and distant metastasis-survival, these markers were implemented in prognostic nomograms developed by Kattan et al.^3^ from Memorial Sloan Kettering Cancer Center (MSKCC) and further refined by Callegaro et al.^4^. However, due to the heterogeneity in the clinical and pathological characteristics, the accurate prediction of prognosis in patients with STS still remains challenging. Furthermore, none of the available prognostic tools includes serum markers, which have already been proven to predict outcome in STS patients^5,6^. Hence, there is still a strong need for readily available and economically feasible markers, that further help to identify patients at higher risk for tumor recurrence.

The interaction between DM and cancer is investigated with great effort. Insulin and the insulin like growth factors (IGF) have mitogenic effects on various types of cells, including tumor cells^7,8^. This leads to increased cell proliferation, inhibition of apoptosis and activation of protein synthesis^9^. Furthermore, epidemiologic data shows, that the insulin/IGF system might play an important role in enhancing cancer growth in patients with insulin resistance^10,11^. On the other hand, cancer and DM commonly induce chronic inflammatory processes. Inflammation causes hypersecretion of proinflammatory cytokines leading to alterations in the tumor microenvironment^12^. The consecutive immigration of leukocytes, especially tumor associated macrophages^13^, results in an increase of angiogenesis and tumor proliferation^14^. While the exact pathophysiological mechanisms underlying the complex interaction of DM and tumor cells are still not exactly clarified, there is an increasing interest for clinical interpretation of these associations and the investigation as a clinical useful marker^15–17^. Growing evidence shows that DM has been identified as a negative prognostic marker for clinical outcome in several tumor entities^15,16,18,19^. However, the role of DM and pre-surgery glucose levels on survival in STS is unclear and not thoroughly investigated.

In this study, we evaluated the effect of DM and pre-surgery blood glucose levels on CSS, OS and DFS in patients with localized STS.

## Patients and methods

### Study participants

We performed a retrospective analysis of 475 patients with a histologically confirmed diagnosis of STS. All patients underwent curative tumor resection between 1998 and 2016 at the Department of Orthopedics and Trauma, Medical University of Graz, Austria. Patients with metastases at the time of diagnosis were excluded from the study.

All patients were included in the follow-up program of the Department of Orthopedics and Trauma and the Division of Clinical Oncology, Department of Medicine, Medical University of Graz, providing follow-up examinations in regular intervals (3 months intervals in years 1–3, 6 months intervals in years 4–5, and 12 months intervals in years 6–10 after diagnosis). Primary predictor variables included pre-existing DM and mean pre-surgery glucose levels. The information about pre-existing DM was extracted from the medical records of patients, mean pre-surgery blood glucose levels were obtained from pre-operative laboratory tests taken within the two weeks before surgery. Clinicopathological data including demographic variables, tumor characteristics and adjuvant treatment were retrospectively obtained from the patient´s history. Follow-up investigations included clinical check-up and radiological analyses (computed tomography, magnetic resonance imaging, abdominal ultrasound and chest X-ray). For the present study, all histological specimens were centrally reviewed by an independent experienced soft tissue pathologist (B. LA.). All sarcomas were diagnosed according to the current WHO classification of soft tissue and bone tumors^20^. Tumors were either graded according to the French Federation of Cancer Centers Sarcoma Group (FNCLCC) grading system^21^ or tumor grade was defined by tumor entity. Malignant fibrous histiocytomas had been re-classified according to the current diagnostic criteria^20,22^. This study was approved by the Ethics Committee of the Medical University of Graz (EK 30-436 ex 17/18, ethikkommission@medunigraz.at). All methods were performed in accordance with the relevant local and national guidelines and regulations.

### Statistical analysis

The primary endpoint of this study was CSS, which was calculated from the date of diagnosis to the date of cancer-related death. Secondary endpoints included OS (time between diagnosis and death of any cause) and DFS (time between diagnosis and local recurrence or occurrence of distant metastases). Predictor variables included pre-existing DM and mean pre-surgery glucose levels. The mean pre-surgery glucose was calculated by taking the average of random or fasting blood glucose values measured within two weeks before surgery. We extracted the data in Microsoft Excel 2016 and imported it into Stata 15.0 for statistical analysis. We summarized outcome and predictor variables as frequencies (%) and means (± standard deviations [SD]) as appropriate, overall and by DM status. We used Chi-square tests, Fischer Exact tests or unpaired t-tests to compare qualitative and quantitative variables with DM status as appropriate. We presented CSS, overall and by DM, as cumulative incidence function (CIF) plots and used Gray’s test to compare CIFs by DM. We presented OS and DFS as Kaplan Meier plots and used log rank tests to compare survival curves by DM status.

We performed Fine and Gray competing risks regression to assess the association of DM and mean pre-surgery glucose with CSS. We performed simple and multiple Cox-proportional hazard regression to assess the association of DM with outcomes in all patients and mean pre-surgery glucose with outcomes in non-diabetic patients. In multiple Cox and competing risk regression analysis, we adjusted for age, sex, resection margins, tumor size, tumor grade, tumor depth, adjuvant chemotherapy and adjuvant radiation therapy. We chose the *p*-value < 0.05 and the confidence interval (CI) of 95% to determine statistical significance.

### Ethical approval

Written informed consent was not obtained from individual patients, because the Ethics Committee of the Medical University of Graz (EK 30-436 ex 17/18, ethikkommission@medunigraz.at) specifically granted a “waiver of consent” for this retrospective database study. All methods were performed in accordance with the relevant local and national guidelines and regulations. All investigations have been in accordance with the principles embodied in the declaration of Helsinki.

## Results

**Figure 1:**
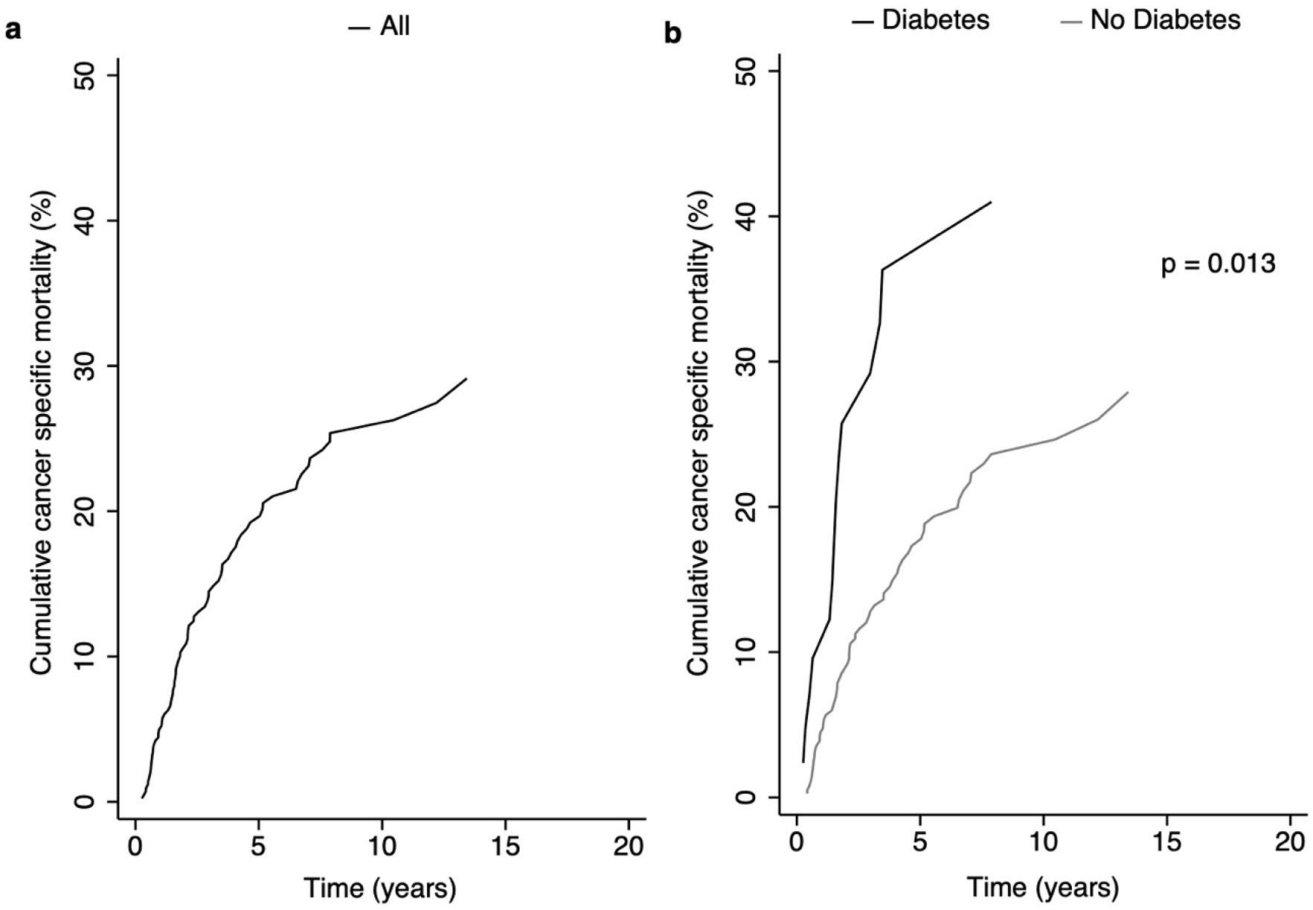
Cumulative cancer specific mortality, overall (**a**) and by diabetes mellitus (**b**).

The median follow-up time was 34 months (1–214 months). The mean age at the time of surgery was 60.1 ± 17.5 years. Table 1 summarizes the characteristics of STS patients overall and in patients with and without DM. A total of 44 patients (9.3%) had preexisting DM. To strengthen the preexistence of DM we extracted the use of antidiabetic drugs from these patients’ medical records. Of all patients with DM, 31 (70.5%) had documented use of at least one of the following antidiabetic drugs at the time of surgery. The most common prescribed drug was metformin (14/31) and secretagogue based therapy (sulfonylureas and repaglinide, 14/31) followed by insulin (10/31), dipeptidyl peptidase-4 inhibitors (DPP4i, 3/31), glucagon-like peptide-receptor agonists (GLP1a, 1/31) and alpha glucosidase inhibitors (AGI, 1/31). The mean pre-surgery glucose of all participants was 106.7 ± 28.4 mg/dl. Of the 475 STS patients, 128 (26.9%) developed an overall disease recurrence, with 45 (9.5%) patients showing a local recurrence and 104 (21.9%) presenting with distant metastases, including those patients who had both local- and distant recurrence. Overall, 129 (27.2%) patients died during follow-up and advanced disease was the cause of death in 79 (16.7%) patients (Fig. 1a). Age (p < 0.001) and mean pre-surgery glucose level (p < 0.001) were significantly higher in patients with DM compared to those without DM (Table 1). Furthermore, adjuvant chemotherapy was administered significantly more often in patients without diabetes as compared to those with known DM (p = 0.017) (Table 1). With respect to gender, tumor location, tumor size, tumor depth, tumor grading, histology, resection margins and adjuvant radiation, there was no statistically significant difference between the two patient groups (Table 1).

**Table 1:** Characteristics of patients, overall and by diabetes mellitus (N = 475).

Variable	N	All (n = 475)	DM (n = 44)	No DM (n = 431)	*P*-value
**Age (years)**	475	60.2 ± 17.5	73.6 ± 9.9	58.9 ± 17.5	< 0.001
Age < 40		71 (14.9%)	0 (0.0%)	71 (16.5%)	< 0.001
Age 40–59		141 (29.7%)	5 (11.4%)	136 (31.5%)	
Age ≥ 60		263 (55.4%)	39 (88.6%)	224 (52.1%)	
**Gender**	475				
Male		259 (54.5%)	21 (47.7%)	238 (55.2%)	0.342
Female		216 (45.5%)	23 (52.3%)	193 (44.8%)	
**Serum pre-surgery glucose [mg/dl]**	395	106.7 ± 28.4	149.2 ± 47.3	101.8 ± 20.8	< 0.001
**Tumor location**	474				
Head/neck		7 (1.5%)	1 (2.3%)	6 (1.4%)	0.784
Thoracic/trunk		38 (8.0%)	26 (59.1%)	272 (63.3%)	
Retro/intra-abdominal		7 (1.5%)	0 (0.0%)	7 (1.6%)	
Upper extremity		124 (26.2%)	5 (11.4%)	33 (7.7%)	
Lower extremity		298 (62.9%)	12 (27.3%)	112 (26.1%)	
**Tumor size (cm)**	462	8.7 ± 6.0	9.1 ± 5.6	8.6 ± 6.0	0.592
**Tumor size—categories**	462				
< 5 cm		135 (29.2%)	9 (20.9%)	126 (30.1%)	0.448
5–10 cm		188 (40.7%)	20 (46.5%)	168 (40.1%)	
> 10 cm		139 (30.1%)	14 (32.6%)	125 (29.8%)	
**Tumor depth**	472				
Superficial		131 (27.7%)	12 (27.3%)	119 (27.8%)	0.946
Deep		284 (60.2%)	26 (59.1%)	258 (60.3%)	
Both superficial and deep		57 (12.1%)	6 (13.6%)	51 (11.9%)	
**Tumor grading**	441				
G1		91 (20.6%)	7 (15.9%)	84 (21.2%)	0.701
G2		90 (20.4%)	9 (20.5%)	81 (20.4%)	
G3		260 (59.0%)	28 (63.6%)	232 (58.4%)	
**Histology**	475				
Angiosarcoma		6 (1.3%)	0 (0.0%)	6 (1.4%)	0.064
MPNST		11 (2.3%)	1 (2.3%)	10 (2.3%)	
Myxofibrosarcoma		135 (28.4%)	13 (29.5%)	122 (28.3%)	
Synovial sarcoma		34 (7.2%)	0 (0.0%)	34 (7.9%)	
UPS		46 (9.7%)	10 (22.7%)	36 (8.3%)	
Spindle cell sarcoma		9 (1.9%)	0 (0.0%)	9 (2.1%)	
Liposarcoma		106 (22.3%)	11 (25.0%)	95 (22.0%)	
Other		128 (26.9%)	9 (20.5%)	119 (27.6%)	
**Resection margins**	475				
R0		459 (96.6%)	42 (95.5%)	417 (96.7%)	0.650
R1		16 (3.4%)	2 (4.5%)	14 (3.3%)	
**Adjuvant radiation**	448	235 (52.5%)	26 (61.9%)	209 (51.5%)	0.198
**Adjuvant chemotherapy**	475	50 (10.5%)	0 (0.0)	50 (11.6)	0.017

Chi-square and Fischer Exact tests were applied to compare qualitative variables with diabetes status. Unpaired t-tests were applied to compare quantitative variables with diabetes.

*DM* Diabetes mellitus, *MPNST* Malignant, Peripheral Nerve Sheath Tumors, *UPS* Undifferentiated Pleomorphic Sarcoma.

The results for 5- and 10-year OS, DFS, local recurrence-free survival (LRFS) and distant metastasis-free survival (DMFS) in STS patients with and without DM are shown in Table 2.

**Table 2.** 5-year and 10-year survival estimates, overall and by diabetes mellitus (DM).

Variable	5-year survival	10-year survival
	Estimate (95%CI)	Estimate (95%CI)
**Overall survival**		
All	74.5% (69.7–78.7%)	60.0% (53.6–66.0%)
Diabetes	54.1% (36.9–68.4%)	39.3% (21.4–56.8%)
No diabetes	76.8% (71.8–80.1%)	62.4% (55.4–68.6%)
**Disease free survival**		
All	70.1% (65.0–74.6%)	62.3% (55.9–68.0%)
Diabetes	59.4% (41.3–73.6%)	52.4% (31.9–69.5%)
No diabetes	71.2% (65.8–75.8%)	63.2% (56.4–69.2%)
**Local recurrence free survival**		
All	89.0% (85.0–92.0%)	83.2% (77.2–87.7%)
Diabetes	80.3% (61.4–90.6%)	70.9 (43.7–86.6%)
No diabetes	89.9% (85.9–92.8%)	84.3% (78.1–88.9%)
**Distant metastasis free survival**		
All	74.6% (69.6–78.8%)	70.2% (64.4–75.3%)
Diabetes	66.7% (47.9–80.0%)	66.7% (47.9–80.0%)
No diabetes	75.3% (70.1–79.7%)	70.5% (64.3–75.9%)

*CI* Confidence Interval

The cumulative cancer specific mortality was significantly higher in STS patients with DM compared to those without DM (p = 0.013) (Fig. 1b).

**Figure 2:**
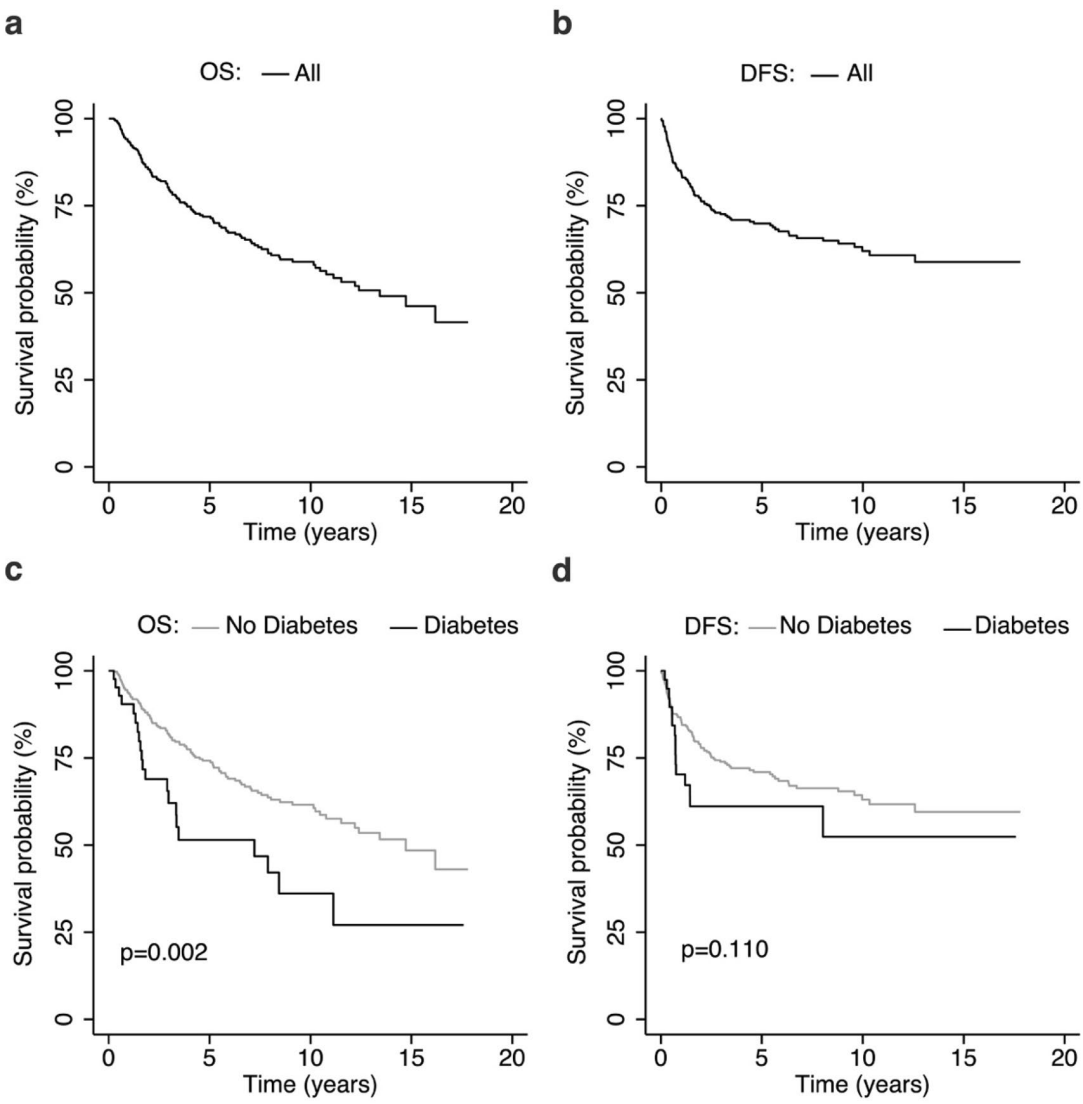
Kaplan Meier plots of overall survival (**a**, **c**) and disease-free survival (**b**, **d**), of all patients and by diabetes mellitus.

Figure 2c shows that OS was significantly lower in patients with DM compared to those without DM (p = 0.002). DFS did not differ significantly between DM and non-DM patients (Fig. 2d).

We performed an unadjusted Cox-hazard proportional regression of OS (Fig. 2a) and DFS (Fig. 2b) and a competing risk regression of CSS with DM and other co-variates in this study cohort (Table 3). We identified DM (SHR 2.14, 95% CI 1.18–3.90, p = 0.013) and high tumor grade (p = 0.03) as poor prognostic factors for CSS. In the adjusted analysis that included DM, age, gender, tumor size, tumor depth, tumor grading, resection margins and adjuvant treatment as co-variates, DM (adjusted SHR 2.33, 95% CI 1.21–4.49, p = 0.012) and high tumor grade (p = 0.010) remained adverse prognostic factors for CSS.

**Table 3:** Unadjusted and adjusted competing risk regression of CSS and Cox-hazard proportional regression of OS and DFS with DM and other co-variates in STS patients.

Variables	CSS				OS				DFS			
	Unadjusted competing risk regression		Adjusted competing risk regression		Unadjusted Cox proportional regression		Adjusted Cox proportional regression		Unadjusted Cox proportional regression		Adjusted Cox proportional regression	
	SHR (95% CI)	*P*	aSHR (95% CI)	*P*	HR (95% CI)	*P*	aHR (95% CI)	*P*	HR (95% CI)	*P*	aHR (95% CI)	*P*
**Diabetes**												
No	1		1		1		1		1		1	
Yes	2.14 (1.18–3.90)	0.013	2.33 (1.21–4.49)	0.012	2.05 (1.28–3.28)	0.003	1.96 (1.17–3.28)	0.010	1.55 (0.90–2.66)	0.113	1.52 (0.86–2.67)	0.149
**Age**												
< 40 years	1		1		1		1		1		1	
40–59 years	0.78 (0.38–1.57)	0.486	0.92 (0.42–2.02)	0.843	0.86 (0.45–1.63)	0.637	1.24 (0.60–2.58)	0.554	1.14 (0.58–2.24)	0.699	1.30 (0.63–2.66)	0.479
≥ 60 years	1.07 (0.57–2.00)	0.842	1.19 (0.54–2.59)	0.665	1.84 (1.05–3.24)	0.034	2.17 (1.10–4.28)	0.025	1.89 (1.03–3.48)	0.040	1.63 (0.81–3.29)	0.172
**Sex**												
Male	1		1		1		1		1		1	
Female	0.69 (0.84–1.09)	0.113	0.67 (0.42–1.09)	0.110	0.91 (0.64–1.28)	0.577	0.94 (0.65–1.37)	0.747	0.86 (0.60–1.24)	0.429	0.96 (0.66–1.41)	0.854
**Tumor size (cm)**	1.02 (0.99–1.05)	0.137	1.02 (0.98–1.05)	0.314	1.02 (0.99–1.05)	0.075	1.03 (0.99–1.06)	0.092	1.04 (1.01–1.06)	0.005	1.03 (1.01–1.07)	0.027
**Tumor depth**												
Superficial	1		1		1		1		1		1	
Deep	1.23 (0.72–2.11)	0.448	1.54 (0.81–2.91)	0.186	0.85 (0.56–1.29)	0.442	0.92 (0.58–1.47)	0.739	1.10 (0.71–1.70)	0.663	0.93 (0.57–1.51)	0.774
Deep + superficial	1.75 (0.86–3.57)	0.123	2.05 (0.97–4.36)	0.060	1.74 (1.04–2.90)	0.035	1.71 (0.97–3.02)	0.065	1.68 (0.95–2.96)	0.076	1.38 (0.75–2.53)	0.293
**Tumor grading**												
G1 + G2	1		1		1		1		1		1	
G3	2.07 (1.28–3.34)	0.003	2.24 (1.21–4.15)	0.010	2.29 (1.55–3.40)	< 0.001	2.22 (1.41–3.49)	0.001	2.59 (1.69–3.96)	< 0.001	2.57 (1.58–4.18)	< 0.001
**Resection margins**												
R0	1		1		1		1		1		1	
R1	0.66 (0.15–2.92)	0.582	0.63 (0.13–2.95)	0.559	1.03 (0.42–2.51)	0.956	0.75 (0.30–1.91)	0.550	1.65 (0.77–3.55)	0.197	1.05 (0.41–2.65)	0.927
**Adj. radiation**												
No	1		1		1		1		1		1	
Yes	1.11 (0.70–1.76)	0.663	0.86 (0.52–1.43)	0.563	1.02 (0.70–1.46)	0.932	0.80 (0.54–1.18)	0.260	1.15 (0.79–1.68)	0.454	0.68 (0.60–1.38)	0.668
**Adj. chemotherapy**												
No	1		1		1		1		1		1	
Yes	1.55 (0.92–2.62)	0.097	1.55 (0.77–3.11)	0.220	1.09 (0.68–1.77)	0.711	1.47 (0.84–2.57)	0.178	1.46 (0.90–2.36)	0.126	1.47 (0.82–2.65)	0.198

*Adj* Adjuvant, *CI* Confidence Interval, *CSS* Cancer Specific Survival, *OS* Overall Survival, *DFS* Disease-free Survival, *HR* Hazard Ratio, *SHR* Sub-hazard Ratio, *aSHR* Adjusted Sub-hazard Ratio.

With regard to OS, in addition to DM and tumor grade (DM: HR 2.05 (95% CI 1.28–3.28), p = 0.03; tumor grade: p < 0.001), age (p = 0.034) and extensive tumor expansion, from deep to superficial locations (p = 0.035) were found to have a significantly negative prognostic impact on OS. In the adjusted model, DM (adjusted HR 1.96, 95% CI 1.17–3.28, p = 0.010), age (p = 0.025) and high tumor grade (p = 0.001) remained significantly associated with adverse outcome, while tumor location showed no significance.

Furthermore, age (p = 0.040), large tumor size (p = 0.005) and high tumor grading (p < 0.001) were associated with a inferior DFS. However, DM showed no association with DFS. Gender, resection margins and adjuvant treatment were not associated with clinical outcome in this analysis (Table 3). Tumor size (p = 0.027) and tumor grading (p < 0.001) remained negative prognostic factors for DFS, while age did not show a significant association anymore (Table 3).

In unadjusted competing risk regression of CSS with pre-surgery glucose and other co-variates in non-DM STS patients, high tumor grading (p = 0.002) and adjuvant chemotherapy (p = 0.027) were identified as negative prognostic factors (Table 4). Unadjusted Cox-hazard proportional regression of OS and DFS with pre-surgery glucose and other co-variates in non-DM STS patients revealed an association of high tumor grading (p < 0.001), OS and inferior DFS for patients with a large tumor size (p = 0.004) and a high tumor grade (p < 0.001) (Table 4). In these analyses, pre-surgery glucose, age, gender, tumor location, resection margins and adjuvant treatment were not associated with clinical outcome. The adjusted analysis included pre-surgery glucose, age, gender, tumor size, tumor depth, tumor grading, resection margins and adjuvant treatment and showed a significant association between high tumor grading (p = 0.015) and adverse clinical outcome for CSS. Decreased OS correlated with age (p = 0.033) and high tumor grading (p < 0.001). For DFS, the only independent negative prognostic factor was high tumor grading (p < 0.001) (Table 4).

**Table 4:** Unadjusted and adjusted competing risk regression of CSS and Cox-hazard proportional regression of OS and DFS with pre-surgery glucose and other co-variates in non-DM STS patients.

Variables	CSS				OS				DFS			
	Unadjusted competing risk regression		Adjusted competing risk regression		Unadjusted Cox proportional regression		Adjusted Cox proportional regression		Unadjusted Cox proportional regression		Adjusted Cox proportional regression	
	SHR (95% CI)	*P*	aSHR (95% CI)	*P*	HR (95% CI)	*P*	aHR (95% CI)	*P*	HR (95% CI)	*P*	aHR (95% CI)	*P*
**Pre-surgery glucose**^a^	0.94 (0.80–1.09)	0.392	0.93 (0.76–1.15)	0.510	0.99 (0.89–1.09)	0.814	0.94 (0.82–1.08)	0.382	1.03 (0.93–1.13)	0.574	1.02 (0.90–1.15)	0.786
**Age**												
< 40 years	1		1		1		1		1		1	
40–59 years	0.74 (0.36–1.53)	0.423	0.88 (0.37–2.08)	0.775	0.85 (0.44–1.64)	0.627	1.37 (0.63–3.00)	0.429	1.16 (0.59–2.29)	0.662	1.37 (0.63–2.98)	0.428
≥ 60 years	0.92 (0.48–1.77)	0.808	1.17 (0.49–2.79)	0.719	1.67 (0.94–2.96)	0.081	2.22 (1.06–4.63)	0.033	1.81 (0.97–3.35)	0.061	1.38 (0.64–2.97)	0.413
**Sex**												
Male	1		1		1		1		1		1	
Female	0.74 (0.45–1.21)	0.231	0.73 (0.40–1.35)	0.323	0.90 (0.62–1.32)	0.608	0.95 (0.61–1.47)	0.828	0.83 (0.56–1.23)	0.350	1.02 (0.65–1.59)	0.934
**Tumor size (cm)**	1.02 (0.99–1.05)	0.208	1.01 (0.97–1.05)	0.642	1.03 (1.00–1.06)	0.055	1.03 (0.99–1.06)	0.095	1.04 (1.01–1.07)	0.004	1.03 (0.99.–1.06)	0.096
**Tumor depth**												
Superficial	1		1		1		1		1		1	
Deep	1.19 (0.66–2.14)	0.570	1.36 (0.64–2.87)	0.425	0.79 (0.50–1.24)	0.298	0.71 (0.41–1.23)	0.225	1.17 (0.73–1.88)	0.521	0.84 (0.47–1.49)	0.546
Deep + superficial	1.53 (0.69–3.40)	0.291	1.62 (0.65–4.08)	0.302	1.57 (0.90–2.74)	0.112	1.35 (0.70–2.59)	0.363	1.77 (0.96–3.26)	0.068	1.26 (0.62–2.54)	0.522
**Tumor grading**												
G1 + G2	1		1		1		1		1		1	
G3	2.43 (1.41–4.21)	0.002	2.58 (1.20–5.56)	0.015	2.86 (1.82–4.50)	< 0.001	2.98 (1.70–5.23)	< 0.001	2.83 (1.78–4.50)	< 0.001	3.49 (1.93–6.33)	< 0.001
**Resection margins**												
R0	1		1		1		1		1		1	
R1	0.86 (0.19–3.93)	0.847	1.09 (0.22–5.45)	0.918	0.99 (0.36–2.70)	0.991	0.87 (0.26–2.90)	0.824	1.96 (0.91–4.23)	0.085	1.99 (0.75–5.32)	0.169
**Adj. radiation**												
No	1		1		1		1		1		1	
Yes	1.20 (0.72–2.00)	0.482	0.95 (0.52–1.65)	0.793	0.99 (0.66–1.47)	0.941	0.78 (0.50–1.22)	0.285	1.31 (0.87–1.97)	0.187	1.01 (0.62–1.62)	0.977
**Adj. chemotherapy**												
No	1		1		1		1		1		1	
Yes	1.84 (1.07–3.16)	0.027	1.31 (0.59–2.87)	0.507	1.27 (0.78–2.07)	0.333	1.24 (0.68–2.27)	0.484	1.61 (0.99–2.62)	0.057	1.22 (0.64–2.34)	0.538

*Adj* Adjuvant, *CI* Confidence Interval, *CSS* Cancer Specific Survival, *OS* Overall Survival, *DFS* Disease-free Survival, *HR* Hazard Ratio, *SHR* Sub-hazard Ratio, *aSHR* Adjusted Sub-hazard Ratio.

^a^Unit change of 5 mg/dl.

## Discussion

In the present study, we demonstrated that DM represents an independent prognostic marker for shorter CSS and OS in a large cohort of STS patients after curative resection. However, the inclusion of mean pre-surgery serum glucose values to well-established prognostic factors in STS patients did not add prognostic information in people without established DM.

The negative impact of DM on clinical outcome was reported in many tumor entities, including breast cancer, endometrial cancer and colon cancer^15–17^. Data regarding the influence of DM on clinical outcome in STS patients is sparse. Kang et al. evaluated the prognostic impact of comorbidities including DM on clinical outcome in 349 STS patients that had undergone surgery for high-grade localized disease and found that the presence of comorbidity was independently associated with poor local recurrence-free survival (LRFS) and disease-specific survival (DSS)^23^. However, as the effect of DM on survival was not separately analyzed in this study, the influence of DM on clinical outcome in STS patients remains unclear^23^. To the best of our knowledge, the impact of pre-existing DM or pre-surgery serum glucose levels on survival of patients with STS has not been studied yet. Due to the rarity and heterogeneity of STS, which account for approximate 1% of all adult malignancies, the treatment of these tumors presents a major challenge in the daily clinical routine^24^. Therefore, it is of utmost importance to find markers that will guide clinical decision making.

The present study shows that in patients with localized STS, pre-existing DM is a prognostic marker for inferior outcome. As expected, the OS was significantly lower in STS patients with DM. However, we also found, that in patients with DM, the CSS was significantly reduced. This association could also be found in multivariable modeling, even after adjusting for various established risk factors, indicating that the presence of DM is an independent prognostic marker of worse CSS. The circumstance that the association between DM and adverse DFS was not statistically significant is likely due to the relatively small sample size of patients with DM and the low event rate. However, as the Kaplan–Meier curve (Fig. 2D) shows a trend towards decreased DFS, future studies with more participants might uncover a relation between DM and cancer recurrence. The finding that the administration of adjuvant chemotherapy was associated with an inferior CSS in non-DM patients in unadjusted analysis can be explained by the fact that all of these patients had tumors with poor prognosis as estimated by established markers like tumor size greater than 5 cm, deep tumor location and high tumor grade (G2/3). After adjusting for these approved prognostic factors, this association did not remain.

Inferior OS in STS patients with DM can be explained merely by the coexistence of two diseases. However, decreased CSS suggests that the relation between DM and STS nullifies pure cumulative effects of comorbidities, thus suggesting that DM per se is a negative prognostic marker for tumor specific survival in patients with STS.

It has long been recognized that tumor cells metabolize glucose in an extensive way, as postulated in 1924 by Warburg et al.^25^ Unlike most tissues, tumor cells seem to metabolize glucose via aerobic glycolysis instead of oxidative phosphorylation, even in an oxygen rich environment^26^. It seems paradoxical that tumor cells switch to a biochemically less energy efficient metabolic pathway. One possible explanation is that for rapid cell proliferation like in cancer cells, it is important to provide large amounts of lipids, nucleotides and amino acids which can be synthesized from glucose^26^. Aerobic glycolysis creates only 2 molecules of ATP per molecule of glucose whereas oxidative phosphorylation creates 36 molecules of ATP per molecule of glucose. The presumed lack of ATP could be compensated by the continual uptake of glucose from the bloodstream^26^. This massive cancer glucose consumption can be used for diagnostic purposes via FDG-PET scans. In STS patients receiving neoadjuvant chemotherapy FDG-PET allows an even more accurate prediction of the histopathologic response than change in tumor size does^27^.

Despite this plausible biologic rationale, we could not find a significant association of elevated mean pre-surgery blood glucose levels with inferior OS, CSS, and DFS. This could likely be due to the noisiness of the glucose values. All of the patients have been in a pre-operative setting and the glucose levels were assessed at various times of the day. As we collected retrospective glucose values, there was no way to determine whether the measured glucose values were collected during fasting or non-fasting states. In order to account for these differences, we took the mean of all available pre-surgery values from a time frame of 2 weeks before surgery. Previous studies have already used this method to examine the impact of elevated glucose levels on cancer progression^28–31^. For example Derr et al. found a significant association between inferior outcome and elevated mean glucose values in patients with glioblastoma^28^. In their study, the glucose values not only included measures from fasting and non-fasting states, but also measurements from the time period between diagnosis and censor date^28^. This suggests major bias, as it is known that critically ill patients usually develop hyperglycemia at the end of life^32^.

A potential explanation for the discrepancy of DM being associated with inferior outcome while we could not find this correlation with elevated mean glucose, might be due to the DM associated tissue- and organ damage in these patients. Additionally, patients usually are monitored more closely for hyperglycemia in the preoperative timeframe and hence might be under more intense glycemia control. Also, the stress induced by the cancer diagnosis as well as the hospital stay itself might have influenced glucose values in these patients^33,34^. Although this current study was not able to find an association between mean glucose levels and outcome, prospectively and standardized blood glucose assessments are needed in order to truly investigate the relevance and clinical impact of blood glucose levels as a prognostic marker in STS patients. Instead of a mean glucose value, future studies could investigate the impact of the hemoglobin A1C as a marker for elevated glucose values over a period of time. HbA1C has the advantage of being an indicator of elevated blood glucose values over a longer period of time^35^. We did not investigate the impact of HbA1C as a prognostic marker due to the lack of this value in most of our patients.

Another limitation of the present study is the limited availability of information regarding other factors which might be responsible for worse prognosis and survival outcomes in the current sample. These factors include obesity, diet, physical activity, smoking status and medications with influence on serum glucose levels. Also, we were not able to support the diagnosis of preexisting DM by extracting data on the use of antidiabetic drugs in every single DM patient. While we adjusted our analysis for the significant baseline differences (age and adjuvant chemotherapy) in the DM and non-DM patients, there still might be unmeasured impact on outcome like undocumented organ damage or yet undiagnosed comorbidities. However, the strength of this study is the large sample size and the long follow-up period.

In conclusion, this is the first study indicating that DM is an independent predictor of adverse clinical outcome in STS patients who underwent curative surgical resection. Large scale prospective studies are warranted to confirm our results.
